# Lauric acid modulates the cyclooxygenases and nitric oxide pathways and reduces oxidative stress in preventing tracheal hyperresponsiveness in asthmatic Wistar rats

**DOI:** 10.3389/fphar.2025.1657799

**Published:** 2026-01-05

**Authors:** Indyra Alencar Duarte Figueiredo, Alissa Maria de Oliveira Martins, Alexya Mikelle Teixeira Cavalcanti, Jayne Muniz Fernandes, Ludmila Emilly da Silva Gomes, Gabriel Nunes Machado de Oliveira, Lucas Nóbrega de Oliveira, Isabela Motta Felício, Adriana Maria Fernandes de Oliveira Golzio, Adriano Francisco Alves, Luiz Henrique César Vasconcelos, Fabiana de Andrade Cavalcante

**Affiliations:** 1 Functional Pharmacology Laboratory Professor George Thomas, Drug and Medicine Research Institute, Federal University of Paraíba, João Pessoa, Paraíba, Brazil; 2 Post-graduate Program in Natural and Synthetic Bioactive Products, Federal University of Paraíba, João Pessoa, Paraíba, Brazil; 3 Department of Food Technology, Center for Technology and Regional Development, Federal University of Paraíba, João Pessoa, Paraíba, Brazil; 4 Department of Biomedical Sciences, Health Sciences Center, Federal University of Paraíba, João Pessoa, Paraíba, Brazil

**Keywords:** dodecanoic acid, ovalbumin, asthma, trachea, pulmonary homogenate, oxidative stress

## Abstract

Lauric acid, or dodecanoic acid, a medium-chain fatty acid, prevents alterations in pulmonary ventilation and tracheal hyperresponsiveness in Wistar rats with allergic asthma induced by ovalbumin (OVA). Therefore, the aim was to evaluate the mechanism of action of lauric acid (LA) in its preventive effect on changes caused by asthma. Rats were randomly divided into a control group (CG), an asthmatic group (AG), and an asthmatic lauric acid 25-mg/kg group (ALA25G). Rats in the AG and ALA25G groups were sensitized and challenged with OVA. For the experimental protocols, the trachea and lungs were isolated after euthanasia. A reduction in the contractile reactivity to CCh was observed in the asthmatic group in the presence of indomethacin, zileuton, L-NAME, apocynin, and tempol, inhibitors of COX, 5-LOX, NOS, NADPH oxidase, and a mimetic of superoxide dismutase (SOD), respectively. In the ALA25G, the contractile reactivity was reduced in the presence of indomethacin, L-NAME, and apocynin. Furthermore, an increase in lipid peroxidation (MDA) and nitrite levels and a reduction of reduced glutathione (GSH) levels and SOD activity were observed in the pulmonary homogenate of the AG. Treatment with lauric acid at a dose of 25 mg/kg prevented all of these alterations, except for the reduction in GSH levels. In conclusion, LA reduces tracheal hyperresponsiveness in Wistar rats with allergic asthma by negatively modulating both the COX and NO pathways and oxidative stress imbalance.

## Graphical Abstract

**Figure:**
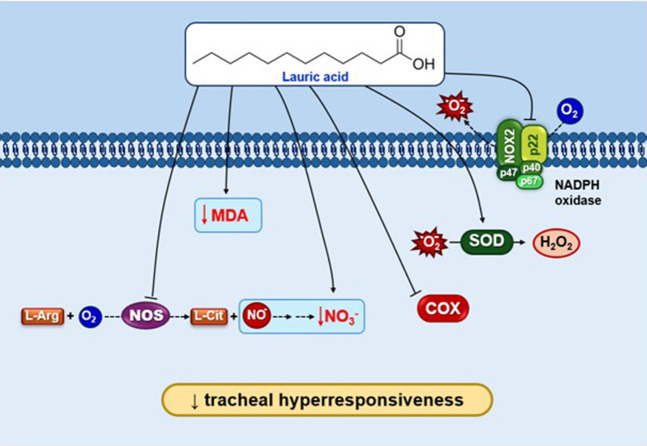
COX, cyclooxygenases; H2O2, hydrogen peroxide; L-Arg, L-arginine; L-Cit, L-citrulline; MDA, malondialdehyde; NO, nitric oxide; NOS, nitric oxide synthase; NOX, NADPH oxidase; “02,” superoxide anion; SOD, superoxide dismutase.

## 1 Introduction

Asthma is a chronic inflammatory airway disease characterized by the combined action of innate and adaptive immune system cells. This leads to hyperresponsiveness, increased mucus production, tissue remodeling, and narrowing of the airway lumen (Hammad and Lambrecht, 2021). Asthma affects more than 300 million individuals worldwide, with prevalence rates ranging from 1% to 29% of the population across different countries (Gina, 2024).

Nitric oxide (NO^·^) has been well described in the literature as an important signaling molecule involved in the pathogenesis of asthma. NO^·^ is generated from L-arginine through the catalytic activity of nitric oxide synthase (NOS) isoenzymes in the presence of various cofactors. Three distinct NOS isoforms are expressed in the lung (Kobzik et al., 1993): neuronal (nNOS or NOS1), inducible (iNOS or NOS2), and endothelial (eNOS or NOS3) (Van Den Berg et al., 2018).

Other mediators that play an important role in airway homeostasis and pathophysiological processes such as asthma are eicosanoids, which include both contractile and relaxing factors of smooth muscle. They originate from the oxidation of arachidonic acid (AA), which is esterified in phospholipids within the cell membrane and released by the action of phospholipase A_2_ (Zhang et al., 2023). Free fatty acids are metabolized by the cyclooxygenase (COX) pathway, generating prostanoids, and the lipoxygenase (LOX) pathway, producing leukotrienes (LTs) (Martin et al., 2016).

Associated with the inflammatory component, asthma is characterized by an increase in oxidative stress, defined as an imbalance between reactive oxygen species (ROS) and reactive nitrogen species (RNS), and the biological system’s ability to detoxify reactive intermediates or repair damage caused by oxidative radicals (Rahal et al., 2014). Reactive species normally function in physiological cellular processes, but at high concentrations, they can damage cellular structures such as carbohydrates, nucleic acids, lipids, and proteins, altering their functions. Under pathological conditions, antioxidant systems may become overwhelmed, leading to an oxidative stress imbalance (Sahiner et al., 2018).

Lauric acid (LA), or dodecanoic acid, is a medium-chain saturated fatty acid and the major component of virgin coconut oil, which, in turn, has shown a preventive effect on changes induced by asthma in animals (Dayrit, 2015; Eyres et al., 2016; Vasconcelos et al., 2020). LA exhibits antihypertensive and vasorelaxant activity in both normotensive and hypertensive rats (Alves et al., 2017), prevents the reduction of cavernous body relaxation in diabetic rats (Olubiyi et al., 2022), reduces inflammation and structural pulmonary changes (Dubo et al., 2019), and mitigates oxidative stress in the lungs of rats with type 2 diabetes mellitus (Augustine et al., 2022). Additionally, LA administered as a single oral dose of 2000 mg/kg did not exhibit acute toxicity in Sprague–Dawley rats (Khan et al., 2020) nor chronic toxicity in albino rats when included in the diet at 10% (Fitzhugh et al., 1960).

In a previous study carried out by our research group, it was found that LA, administered at a dose of 100 mg/kg/day for 28 days, showed low toxicity, considering the absence of mortality or significant changes related to food and water consumption, organ weight, or hematological and biochemical parameters. It was also shown that different doses of LA prevented tracheal hyperresponsiveness induced by carbachol (CCh) and alterations in pulmonary ventilation in Wistar asthmatic rats. Furthermore, among the possible molecular targets of LA’s action identified through *in silico* studies, endothelial nitric oxide synthase (eNOS), inducible nitric oxide synthase (iNOS), cyclooxygenase-2 (COX-2), and 5-lipoxygenase were highlighted, among other proteins (Figueiredo et al., 2025).

Based on this, the objectives of this study were to confirm these potential interaction targets of LA, highlighting its mechanism of action *in vitro*, and to evaluate LA’s role in the oxidative stress imbalance for the prevention of tracheal hyperresponsiveness in asthmatic Wistar rats.

## 2 Materials and methods

### 2.1 Animals

Male Wistar rats (*Rattus norvegicus*) weighing between 250 g and 300 g, at 6–8 weeks of age, sourced from Universidade Estadual da Paraíba (UEPB) and kept at the Animal Production Unit (UPA) of the Instituto de Pesquisa em Fármacos e Medicamentos (IPeFarM) at the Universidade Federal da Paraíba (UFPB), were used. Animals were kept under controlled temperature conditions (22 °C ± 1 °C) and a 12-h light–dark cycle with free access to food and water. Experimental procedures were conducted following the principles of the guidelines for the ethical use of animals in applied etiology studies (Sherwin et al., 2003) and the Brazilian Guide for the Production, Maintenance, or Use of Animals in Educational or Scientific Research Activities by the National Council for the Control of Animal Experimentation (CONCEA) (Brasil, 2016). Experimental procedures were approved by the Animal Use Ethics Committee (CEUA) of UFPB (n° 9310040522).

### 2.2 Chemicals

Sodium chloride (NaCl), potassium chloride (KCl), magnesium sulfate (MgSO_4_), potassium phosphate (KH_2_PO_4_), calcium chloride (CaCl_2_), glucose, sodium bicarbonate (NaHCO_3_), hydrochloric acid (HCl), and sodium hydroxide (NaOH) were obtained from Êxodo Científica (Sumaré, Brazil).

Lauric acid, aluminum hydroxide (Al(OH)_3_), ovalbumin (OVA) (grade II and V), carbamylcholine hydrochloride (CCh), apocynin, Nω-nitro-L-arginine methyl ester hydrochloride (L-NAME), indomethacin, tempol, thiobarbituric acid, trichloroacetic acid, sulfanilamide, N-(1-naphthyl) ethylenediamine hydrochloride, phosphoric acid, 5,5′-dithio-bis(2-nitrobenzoic acid), phosphate buffer, hydrochloric acid, ethylenediaminetetraacetic acid (EDTA), L-methionine, n-butanol, nitroblue tetrazolium, and riboflavin were obtained from Sigma-Aldrich (São Paulo-SP, Brazil).

Zileuton was purchased from Cayman Chemical (Ann Arbor, Michigan, United States). Tween^®^ 80 was obtained from Fischer BioReagents. Ketamine and xylazine were purchased from Syntec (Barueri, São Paulo, Brazil). The carbogenic mixture (95% O_2_ and 5% CO_2_) was purchased from White Martins (Brazil).

### 2.3 Equipment

To record isometric contractions, the organs were suspended in isolated organ baths (6 mL), model BOI-04, and connected to isometric force transducers, model TIM 05, coupled to an amplifier model AECAD04F. This, in turn, was connected to a digital acquisition system, with AQCAD software version 2.5.0 for data acquisition and ANCAD for analysis. The system contained a thermostatic pump, model BT 60, that controlled the temperature of the tanks. All equipment was purchased from AVS Projetos (São Paulo, Brazil).

A refrigerated microcentrifuge, model LIF500R (LabinFarma Scientific, Piracicaba-SP, Brazil), was used to centrifuge the samples. Absorbance measurements were performed using a microplate reader, model MR9600 (Accuris Instruments, New Jersey, United States).

### 2.4 Experimental groups

Rats were randomly divided into five experimental groups, with five male rats each. The control group (CG) was not sensitized and was treated with NaCl 0.9% + Tween^®^ 80; the asthmatic group (AG) was sensitized with OVA and treated with NaCl 0.9% + Tween^®^ 80; and the asthmatic lauric acid 25-mg/kg group (ALA25G) was sensitized with OVA and treated with 25 mg/kg of lauric acid + Tween^®^ 80.

The dose of lauric acid chosen for this study was based on results obtained previously (Figueiredo et al., 2025).

### 2.5 Asthma induction

For the sensitization protocol, on days 1–3 of the experiment, the animals received intraperitoneal (i.p.) injections of 1 mg/kg/day of ovalbumin (OVA) (grade V) solubilized in sterile NaCl 0.9% using 100 mg/mL of aluminum hydroxide (Al(OH)_3_) as an adjuvant. On days 6, 9, 12, 15, 18, and 21, the animals were individually placed in a closed polyacrylic chamber connected to an ultrasonic nebulizer. They were then challenged with 1% OVA (grade II) for up to 20 min daily. Non-sensitized animals underwent the same process but were administered only sterile NaCl 0.9% for both the i.p. injections and nebulizations.

All the animals were euthanized 24 h after the last challenge with OVA or NaCl 0.9% (day 22). Throughout the asthma induction, the asthmatic group animals received daily doses of lauric acid intragastrically. The animals in the CG received NaCl 0.9% via the same route (Salmon et al., 1999; adapted from Galvão et al., 2017; Figueiredo et al., 2025).

### 2.6 Obtaining tracheal rings

Animals were euthanized with ketamine (180 mg/kg, i.p.) and xylazine (30 mg/kg, i.p.), followed by exsanguination. The trachea was then isolated, dissected, and cut into fragments containing 3 to 4 cartilaginous rings in order to standardize sample size across preparations. These segments were individually suspended in an isolated organ bath (6 mL), containing Krebs nutrient solution with the following composition (in mM): NaCl (118.0), KCl (4.5), MgSO_4_ (5.7), KH_2_PO_4_ (1.1), CaCl_2_ (2.5), glucose (11.0), and NaHCO_3_ (25.0), and adjusted to pH 7.4 (with a solution of HCl or NaOH, 1 N). Preparations were kept at a temperature of 37 °C, aerated with carbogen, under tension of 1 g, and allowed to rest for 60 min, with the Krebs solution being changed every 15 min to avoid the influence of metabolites released by the organ into the medium.

### 2.7 Effect of the changes induced by asthma and lauric acid on lung morphology

Lungs of the animals from the CG, AG, ALA25G, ALA50G, ALA100G, and ADEXAG were collected and immediately fixed in 10% buffered formalin for 72 h. Following fixation, standard histological processing was performed, including dehydration in ascending alcohol solutions (70 °GL to absolute alcohol) for 1 h in each solution. Subsequently, samples were immersed twice in xylene baths for 1 h each.

Tissues were embedded in paraffin and sectioned using a rotary microtome at a thickness of 4 μm. Sections were mounted on histological slides, deparaffinized in xylene for 30 min, hydrated in descending alcohol concentrations (absolute, 90°GL, 80°GL, and 70 °GL) for 25 min, and washed in running water for 5 min, followed by distilled water. Samples were then stained with Harris hematoxylin for 1 min, washed again in distilled water for 5 min, and counterstained with eosin for 3 min.

### 2.8 Investigation of the mechanism of action involved in the changes induced by asthma and lauric acid on the contractile reactivity of rat trachea

Each trachea was set up as described previously. After the 60-min stabilization period, when the baseline remained constant, a control cumulative concentration–response curve to CCh was obtained. After 30 min, the trachea was pre-incubated with indomethacin 10^−5^ M, a COX inhibitor (Hua et al., 1996; Sousa et al., 2010); zileuton 10^−5^ M, a 5-LOX inhibitor (adapted from Malo et al., 1994); L-NAME 3 × 10^−4^ M, a non-selective nitric oxide synthase inhibitor (Sousa et al., 2010); apocynin 10^−4^ M, a NADPH oxidase inhibitor (Shabir et al., 2014); tempol 10^−3^ M, a SOD mimetic (De Boer et al., 2001, adapted from Schnackenberg and Wilcox, 2001). The inhibitors were pre-incubated individually for 30 min, after which a new cumulative concentration–response curve to CCh was induced.

The contractile response of the trachea in the presence or absence of inhibitors was calculated based on the maximum tension (g/f) induced by CCh. Contractile reactivity was evaluated from the E_max_ and *p*EC_50_ values of CCh and compared among the CG, AG, and ALA25G groups, both with and without inhibitors.

### 2.9 Effect of lauric acid on the balance between oxidative stress and antioxidant defenses in the lung homogenate

#### 2.9.1 Determination of lipid peroxidation levels

After euthanasia of the animals, lungs were isolated and kept at −20 °C until the preparation of the homogenate. For this, tissue was weighed, macerated, and homogenized with 10% KCl in a 1:1 ratio. The level of lipid peroxidation in rats was analyzed by measuring thiobarbituric acid-reactive substances (TBARS). Lung homogenate was mixed with trichloroacetic acid (10%) and thiobarbituric acid (0.67%) and then placed in a water bath for 15 min. After this period, n-butanol was added to the solution, and the sample was centrifuged (800 g, 5 min). Thiobarbituric acid-reactive substances were determined by measuring absorbance via spectrophotometry at 535 nm. Results were expressed in nmol of malondialdehyde (MDA)/g of organ weight (Draper and Hadley, 1990). The protocol was performed in duplicate.

#### 2.9.2 Determination of nitrite levels

Nitrite levels in the rat lungs were determined using the Griess reaction (Green and Goldman, 1981; Radenovic and Selakovic, 2005). Lung homogenate was centrifuged (800 g/10 min, 24 °C), and the supernatant was collected. Griess reagent (1% sulfanilamide, 0.1% N-(1-naphthyl)ethylenediamine hydrochloride, 5% phosphoric acid, and distilled water in a 1:1:1:1 ratio) was added and incubated at room temperature for 10 min. Nitrite concentration was expressed in nM of nitrite/g of the organ, and absorbance of the samples was measured using spectrophotometry at 560 nm. The protocol was performed in duplicate.

#### 2.9.3 Determination of reduced glutathione levels

This test was performed according to the reaction of Ellman’s reagent (DTNB - 5,5′-dithiobis(2-nitrobenzoic acid)) with thiol groups. Lung homogenate was diluted in 0.02 M EDTA (10%) and mixed with a trichloroacetic acid solution (50%). Samples were then centrifuged (3000 rpm/15 min). Supernatant was collected and mixed with HCl buffer (0.4 M; pH 8.9) and DTNB (0.01 M). The concentration of GSH (ng of GSH/g of organ) was determined using spectrophotometry at 412 nm (Sedlak and Lindsay, 1968). The protocol was performed in duplicate.

#### 2.9.4 Determination of superoxide dismutase activity

The supernatant was centrifuged (20 min, 12,000 rpm, 4 °C), and the resulting supernatant was analyzed. In a dark chamber, 1 mL of the reagent (50 mM phosphate buffer, 100 nM EDTA, and 13 mM L-methionine, pH 7.8) was mixed with 30 µL of the sample, 150 µL of 75 µM NBT (nitro blue tetrazolium), and 300 µL of 2 µM riboflavin. Tubes containing the resulting solution were exposed to a fluorescent lamp (15 W) for 15 min. The absorbance was measured using spectrophotometry at 560 nm. Results were expressed as the unit of SOD required to inhibit the NBT reduction rate by 50% per µg of protein (U/µg protein) (Beauchamp and Fridovich, 1971). The protocol was performed in duplicate.

### 2.10 Statistical analysis

Results were expressed as the mean and standard error of the mean (S.E.M.) and statistically analyzed using one-way analysis of variance (ANOVA), followed by Tukey’s post-test for multiple comparisons between the experimental groups. The null hypothesis was rejected when *p* < 0.05. All data were analyzed using the GraphPad Prism^®^ program 5.01 (Neubig et al., 2003).

## 3 Results

### 3.1 Effect of lauric acid on anatomopathological changes in the lung parenchyma

**FIGURE 1:**
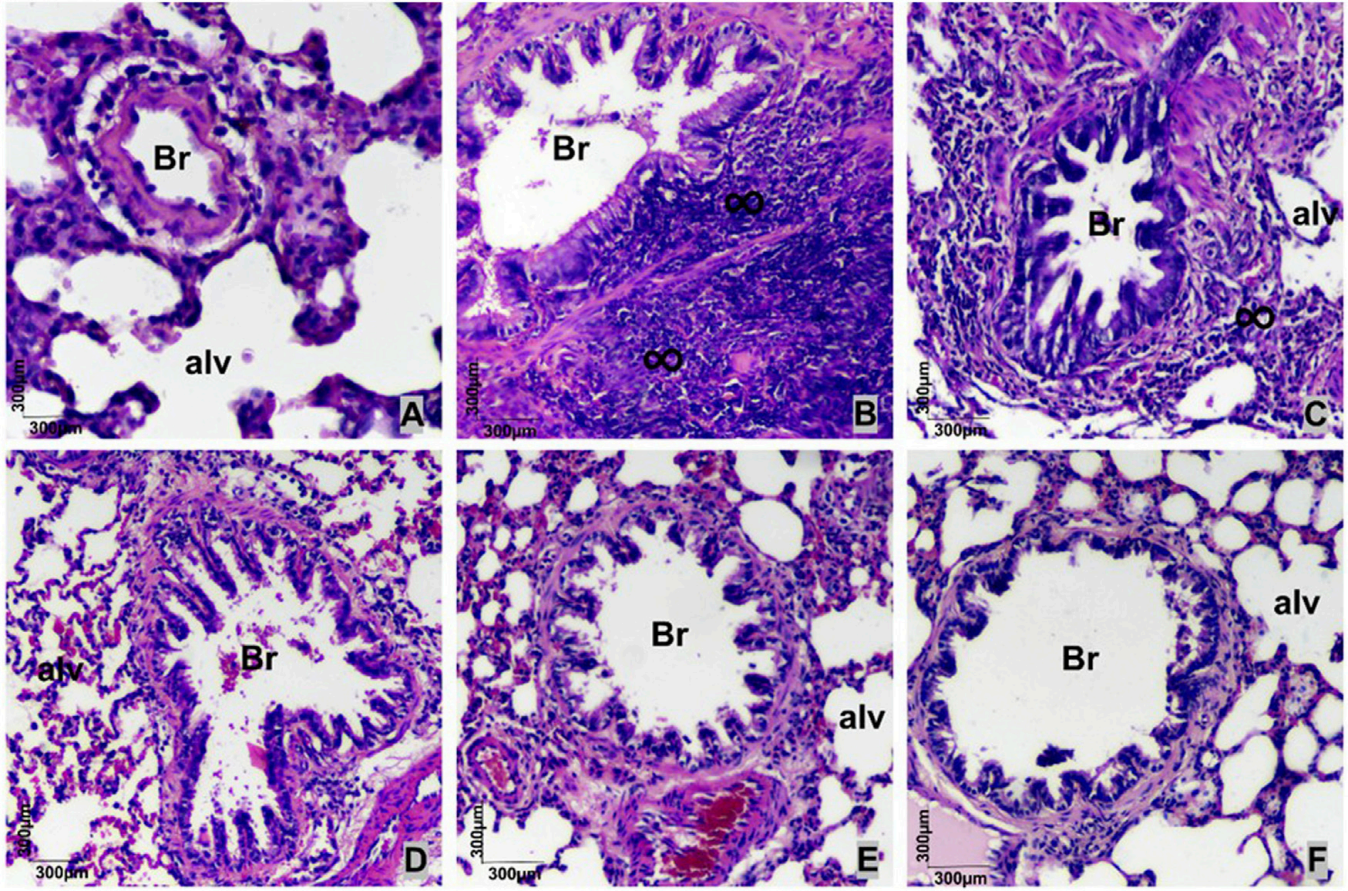
Microphotography of the lung of rats of the CG **(A)**, AG **(B)**, ALA25G **(C)**, ALA50G **(D)**, ALA100G **(E),** and ADEXAG **(F)** stained with hematoxylin–eosin. Alv, alveoli; Br, respiratory bronchioles; ∞ , mononuclear inflammatory infiltrate.

In the histological sections of pulmonary parenchyma stained with hematoxylin–eosin, the CG (Figure 1A) shows bronchioles with preserved epithelium, as well as alveoli with standard morphology. In the AG (Figure 1B), a marked peribronchiolar inflammatory infiltrate is observed, with an increased presence of mononuclear cells and a reduction in the lumen of bronchioles and alveoli. In the ALA25G (Figure 1C), peribronchiolar inflammatory infiltrate is still present, though less intense than in the AG. In contrast, the ALA50G (Figure 1D), ALA100G (Figure 1E), and ADEXAG (Figure 1F) exhibit air spaces with standard morphology, without alterations.

### 3.2 Involvement of the cyclooxygenase pathway in the contractile reactivity of rat trachea

**FIGURE 2:**
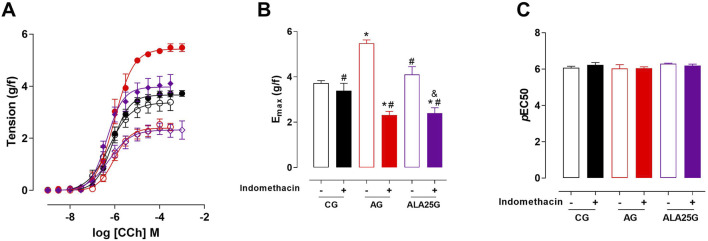
Cumulative concentration–response curves to CCh in rat tracheae of the CG, AG, and ALA25G in the absence (
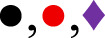
) and presence (
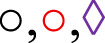
) of indomethacin, respectively **(A)**. E_max_ (g/f) **(B)** and pEC_50_ **(C)** values of CCh in rat tracheae of the CG, AG, and ALA25G in the absence and presence of indomethacin. Symbols and vertical bars represent the mean and S.E.M., respectively. ANOVA one-way followed by Tukey’s *post hoc* test (n = 5). *p < 0.05 (CG vs. AG, AG + indomethacin, and ALA25G + indomethacin); ^#^ *p* < 0.05 (AG vs. CG + indomethacin, AG + indomethacin, ALA25G, and ALA25G + indomethacin); ^&^ *p* < 0.05 (ALA25G vs. ALA25G + indomethacin).

The cumulative concentration–response curve to CCh (10^−9^–10^−3^ M) in the CG was not altered in the presence of indomethacin regarding efficacy or potency. Conversely, the contractile reactivity of the trachea in AG animals was reduced in the presence of the inhibitor, showing lower efficacy but no change in potency. In animals treated with lauric acid at a dose of 25 mg/kg, the cumulative concentration–response curve to CCh showed a reduction in contractile efficacy in the presence of indomethacin, also without changes in potency (Figure 2; Table 1).

**TABLE 1:** E_max_ (g/f) and *p*EC_50_ values of CCh in rat tracheae of the CG, AG, and ALA25G in the absence and presence of indomethacin.

Group	Indomethacin (10^−5^ M)	E_max_ (g/F)	*p*EC_50_
CG	Absence	3.7 ± 0.1	6.0 ± 0.1
	Presence	3.4 ± 0.3	6.2 ± 0.1
AG	Absence	5.5 ± 0.1	6.1 ± 0.2
	Presence	2.3 ± 0.1^a^	6.1 ± 0.1
ALA25G	Absence	4.1 ± 0.3^a^	6.3 ± 0.1
	Presence	2.3 ± 0.2^a^ ^b^	6.1 ± 0.1

Data are expressed as the mean and S.E.M. One-way ANOVA followed by Tukey’s *post hoc* test (n = 5).

^a^ *p* < 0.05.

^b^ *p* < 0.05 (absence vs. indomethacin) in the AG and the ALA25G, respectively.

### 3.3 Participation of the 5-lipoxygenase pathway in the contractile reactivity of rat trachea

**FIGURE 3:**
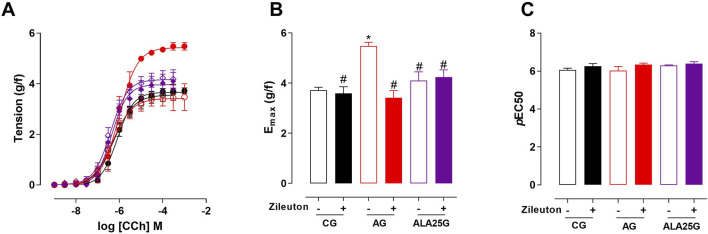
Cumulative concentration–response curves to CCh in rat tracheae of the CG, AG, and ALA25G in the absence (
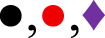
) and presence (
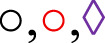
) of zileuton, respectively **(A)**. E_max_ (g/f) **(B)** and pEC_50_ **(C)** values of CCh in rat tracheae of the CG, AG, and ALA25G in the absence and presence of zileuton. Symbols and vertical bars represent the mean and S.E.M., respectively. ANOVA one-way followed by Tukey’s *post hoc* test (n = 5). *p < 0.05 (CG vs. AG); ^#^ *p* < 0.05 (AG vs. AG + zileuton and ALA25G).

The cumulative concentration–response curve to CCh (10^−9^–10^−3^ M) in the CG was not altered in the presence of zileuton regarding efficacy or potency. Conversely, the contractile reactivity of the trachea in AG animals was reduced in the presence of the inhibitor, showing lower efficacy but no change in potency. In animals treated with lauric acid at a dose of 25 mg/kg, the cumulative concentration–response curve to CCh was not altered in the presence of zileuton (Figure 3; Table 2).

**TABLE 2:** E_max_ (g/f) and *p*EC_50_ values of CCh in rat tracheae of the CG, AG, and ALA25G in the absence and presence of zileuton.

Group	Zileuton (10^−5^ M)	E_max_ (g/F)	*p*EC_50_
CG	Absence	3.7 ± 0.1	6.0 ± 0.1
	Presence	3.6 ± 0.3	6.2 ± 0.1
AG	Absence	5.5 ± 0.1	6.1 ± 0.2
	Presence	3.4 ± 0.3^a^	6.3 ± 0.1
ALA25G	Absence	4.1 ± 0.3	6.3 ± 0.1
	Presence	4.2 ± 0.3	6.4 ± 0.1

Data are expressed as the mean and S.E.M. One-way ANOVA followed by Tukey’s *post hoc* test (n = 5).

^a^ *p* < 0.05 (absence vs. zileuton) in the AG.

### 3.4 Participation of the nitric oxide pathway in the contractile reactivity of rat trachea

**FIGURE 4:**
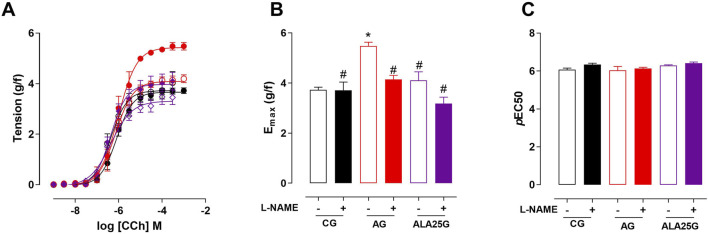
Cumulative concentration–response curves to CCh in rat tracheae of the CG, AG, and ALA25G in the absence (
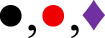
) and presence (
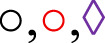
) of L-NAME, respectively **(A)**. E_max_ (g/f) **(B)** and pEC_50_ **(C)** values of CCh in rat tracheae of the CG, AG, and ALA25G in the absence and presence of L-NAME. Symbols and vertical bars represent the mean and S.E.M., respectively. ANOVA one-way followed by Tukey’s *post hoc* test (n = 5). *p < 0.05 (CG vs. AG); ^#^ *p* < 0.05 (AG vs. CG + L-NAME, AG + L-NAME, ALA25G, and ALA25G + L-NAME); ^&^ *p* < 0.05 (ALA25G vs. ALA25G + L-NAME).

The cumulative concentration–response curve to CCh (10^−9^–10^−3^ M) in the CG was not altered in the presence of L-NAME regarding efficacy or potency. Conversely, the contractile reactivity of the trachea in AG animals was reduced in the presence of the inhibitor, showing lower efficacy but no change in potency. In animals treated with lauric acid at a dose of 25 mg/kg, the cumulative concentration–response curve to CCh showed a reduction in contractile efficacy in the presence of L-NAME, also without changes in potency (Figure 4; Table 3).

**TABLE 3:** E_max_ (g/f) and *p*EC_50_ values of CCh in rat tracheae of the CG, AG, and ALA25G in the absence and presence of L-NAME.

Group	L-NAME (3 × 10^−4^ M)	E_max_ (gF)	*p*EC_50_
CG	Absence	3.7 ± 0.1	6.0 ± 0.1
	Presence	3.7 ± 0.3	6.2 ± 0.03
AG	Absence	5.5 ± 0.1	6.1 ± 0.2
	Presence	4.1 ± 0.2^a^	6.1 ± 0.1
ALA25G	Absence	4.1 ± 0.3	6.3 ± 0.1
	Presence	3.2 ± 0.2^b^	6.4 ± 0.1

Data are expressed as the mean and S.E.M. One-way ANOVA, followed by Tukey’s *post hoc* test (n = 5).

^a^ *p* < 0.05.

^b^ *p* < 0.05 (absence vs. L-NAME) in the AG and the ALA25G, respectively.

### 3.5 Participation of NADPH enzymes in the contractile reactivity of rat trachea

**FIGURE 5:**
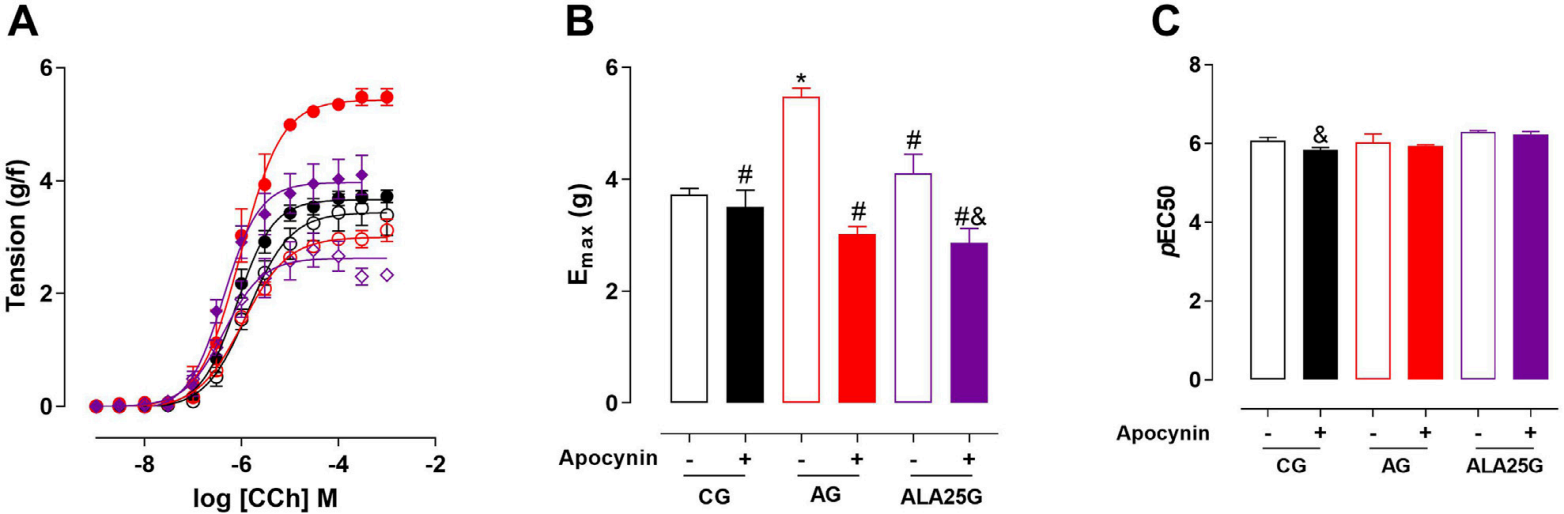
Cumulative concentration–response curves to CCh in rat tracheae of the CG, AG, and ALA25G in the absence (
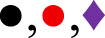
) and presence (
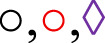
) of apocynin, respectively **(A)**. E_max_ (g/f) **(B)** and pEC_50_ **(C)** values of CCh in rat tracheae of the CG, AG, and ALA25G in the absence and presence of apocynin. Symbols and vertical bars represent the mean and S.E.M., respectively. ANOVA one-way followed by Tukey’s *post hoc* test (n = 5). *p < 0.05 (CG vs. AG); ^#^ *p* < 0.05 (AG vs. CG + apocynin, AG + apocynin, ALA25G, and ALA25G + apocynin); ^&^ *p* < 0.05 (ALA25G vs. ALA25G + apocynin).

The cumulative concentration–response curve to CCh (10^−9^–10^−3^ M) in the CG was not altered in the presence of apocynin regarding efficacy or potency. Conversely, the contractile reactivity of the trachea in AG animals was reduced in the presence of the inhibitor, showing lower efficacy but no change in potency. In animals treated with lauric acid at a dose of 25 mg/kg, the cumulative concentration–response curve to CCh showed a reduction in contractile efficacy in the presence of apocynin (Figure 5; Table 4).

**TABLE 4:** E_max_ (g/f) and *p*EC_50_ values of CCh in rat tracheae of the CG, AG, and ALA25G in the absence and presence of apocynin.

Group	Apocynin (10^−4^ M)	E_max_ (gF)	*p*EC_50_
CG	Absence	3.7 ± 0.1	6.0 ± 0.1
	Presence	3.5 ± 0.3	6.0 ± 0.1
AG	Absence	5.5 ± 0.1	6.1 ± 0.2
	Presence	3.0 ± 0.1^a^	4.6 ± 1.3
ALA25G	Absence	4.1 ± 0.3	6.3 ± 0.1
	Presence	2.9 ± 0.3^b^	6.2 ± 0.1

Data are expressed as the mean and S.E.M. One-way ANOVA followed by Tukey’s *post hoc* test (n = 5).

^a^ *p* < 0.05.

^b^ *p* < 0.05 (absence vs. apocynin) in the AG and the ALA25G, respectively.

### 3.6 Involvement of the SOD enzyme in the contractile reactivity of rat trachea

**FIGURE 6:**
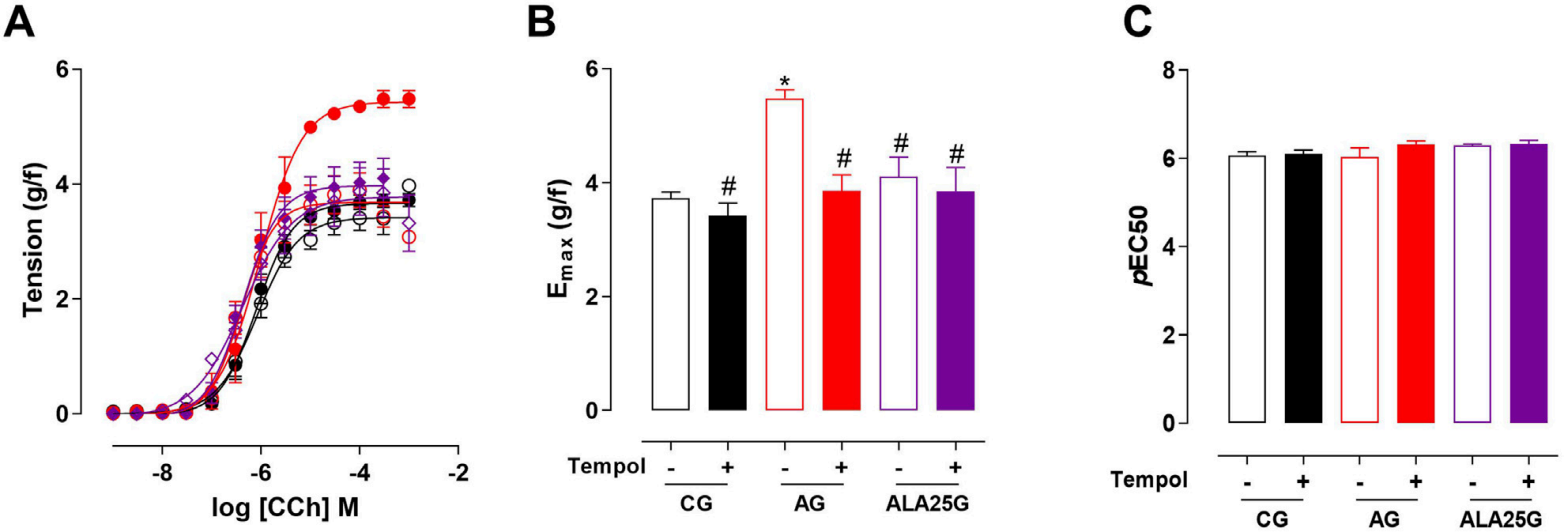
Cumulative concentration–response curves to CCh in rat tracheae of the CG, AG, and ALA25G in the absence (
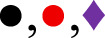
) and presence (
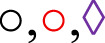
) of tempol, respectively **(A)**. E_max_ (g/f) **(B)** and pEC_50_ **(C)** values of CCh in the rat trachea of the CG, AG, and ALA25G in the absence and presence of tempol. Symbols and vertical bars represent the mean and S.E.M., respectively. ANOVA one-way followed by Tukey’s *post hoc* test (n = 5). *p < 0.05 (CG vs. AG); ^#^ *p* < 0.05 (AG vs. CG + tempol, AG + tempol, ALA25G, and ALA25G + tempol).

The cumulative concentration–response curve to CCh (10^−9^–10^−3^ M) in the CG was not altered in the presence of tempol regarding efficacy or potency. Conversely, the contractile reactivity of the trachea in AG animals was reduced in the presence of the inhibitor, showing lower efficacy but no change in potency. In animals treated with lauric acid at a dose of 25 mg/kg, the cumulative concentration–response curve to CCh was not altered in the presence of tempol (Figure 6; Table 5).

**TABLE 5:** E_max_ (g/f) and *p*EC_50_ values of CCh in rat tracheae of the CG, AG, and ALA25G in the absence and presence of tempol.

Group	Tempol (10^−3^ M)	E_max_ (gF)	*p*EC_50_
CG	Absence	3.7 ± 0.1	6.0 ± 0.1
	Presence	3.4 ± 0.2	6.2 ± 0.1
AG	Absence	5.5 ± 0.1	6.1 ± 0.2
	Presence	3.8 ± 0.3^a^	6.3 ± 0.1
ALA25G	Absence	4.1 ± 0.3	6.3 ± 0.1
	Presence	3.8 ± 0.4	6.3 ± 0.1

Data are expressed as the mean and S.E.M. One-way ANOVA, followed by Tukey’s *post hoc* test (n = 5).

^a^ *p* < 0.05 (absence vs. tempol) in the AG.

### 3.7 Lipid peroxidation levels in the lung homogenate

**FIGURE 7:**
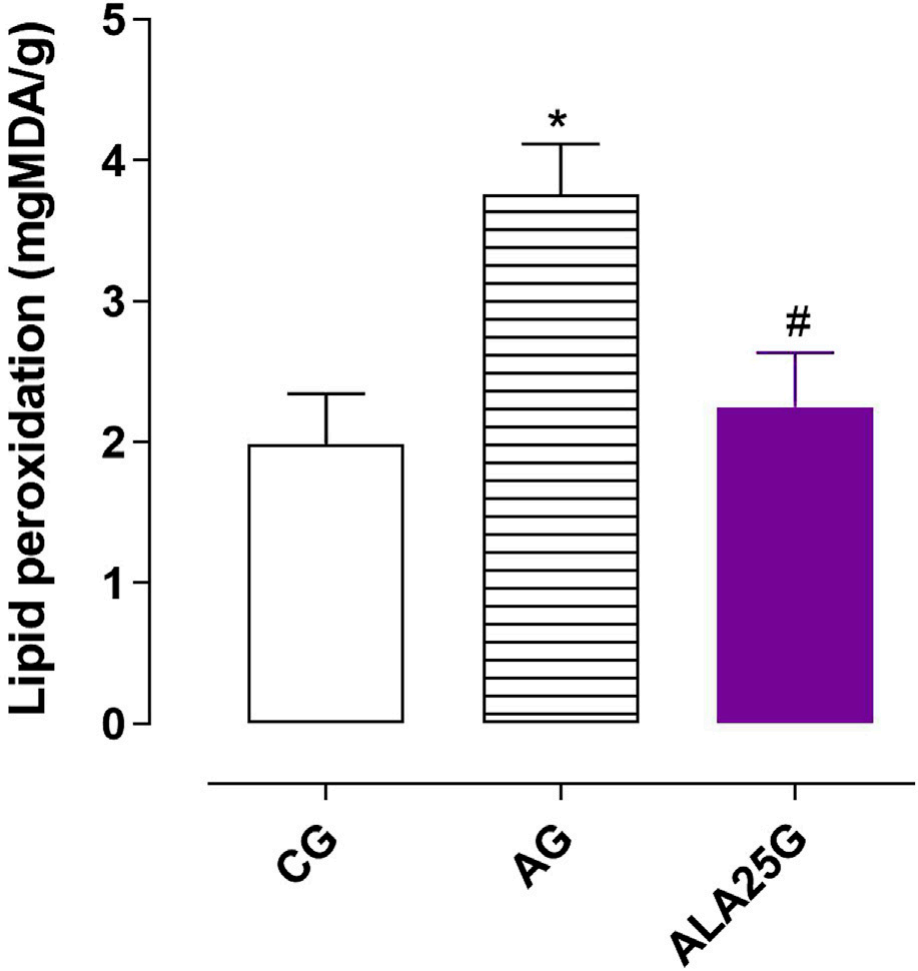
MDA levels (mgMDA/g) in the lung homogenate of rats from the CG, AG, and ALA25G. Symbols and vertical bars represent the mean and S.E.M., respectively. ANOVA one-way followed by Tukey’s *post hoc* test (n = 5). ^*^ *p* < 0.05 (CG vs. AG and ALA25G); ^#^ *p* < 0.05 (AG vs. ALA25G).

The AG showed an increase in MDA concentration (3.8 ± 0.4 mgMDA/g) compared to the CG (1.9 ± 0.3 mgMDA/g). This increase was prevented when the animals were treated with lauric acid at a dose of 25 mg/kg (2.3 ± 0.4 mgMDA/g) (Figure 7).

### 3.8 Nitrite levels in the lung homogenate

**FIGURE 8:**
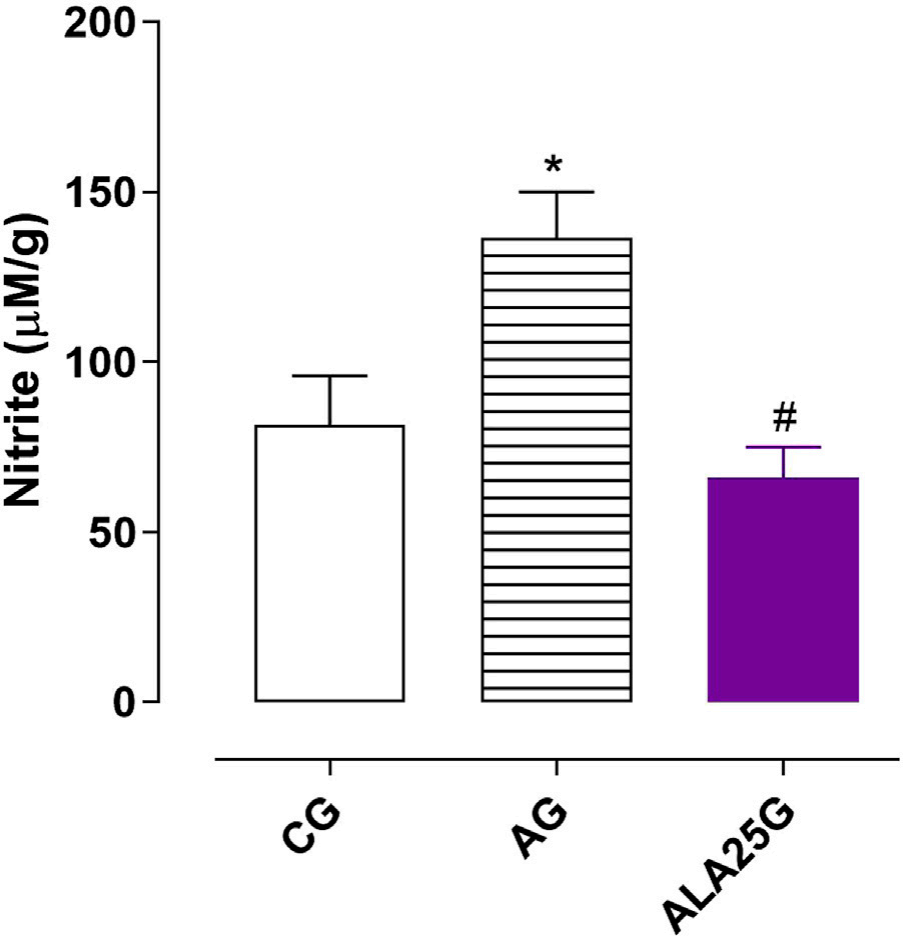
Nitrite levels (μM/g) in the lung homogenate of rats from the CG, AG, and ALA25G. Symbols and vertical bars represent the mean and S.E.M., respectively. ANOVA one-way followed by Tukey’s *post hoc* test (n = 5). ^*^ *p* < 0.05 (CG vs. AG and ALA25G); ^#^ *p* < 0.05 (AG vs. ALA25G).

Nitrite levels were increased in the AG (136.3 ± 13.7 μM/g) compared to the CG (81.5 ± 14.3 μM/g). This increase was prevented when the animals were treated with lauric acid at a dose of 25 mg/kg (65.8 ± 9.1 μM/g) (Figure 8).

### 3.9 GSH levels in the lung homogenate

**FIGURE 9:**
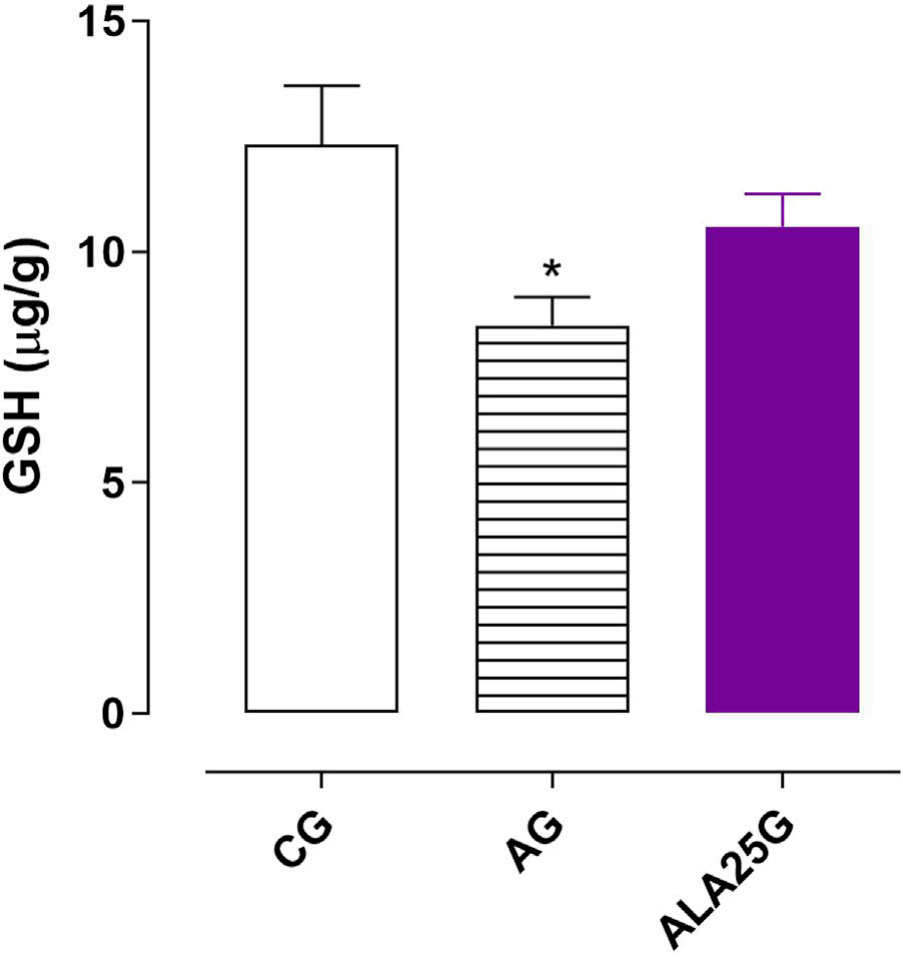
GSH levels (μg/g) in the lung homogenate of rats from the CG, AG, and ALA25G. Symbols and vertical bars represent the mean and S.E.M., respectively. ANOVA one-way followed by Tukey’s *post hoc* test (n = 5). ^*^ *p* < 0.05 (CG vs. AG).

The AG showed a reduction in GSH levels (8.4 ± 0.6 μg/g) compared to the CG (12.3 ± 1.3 μg/g). However, treatment with lauric acid at a dose of 25 mg/kg did not prevent this reduction (10.5 ± 0.7 μg/g), as there was no significant difference between the ALA25G and either the CG or the AG (Figure 9).

### 3.10 SOD activity in the lung homogenate

**FIGURE 10:**
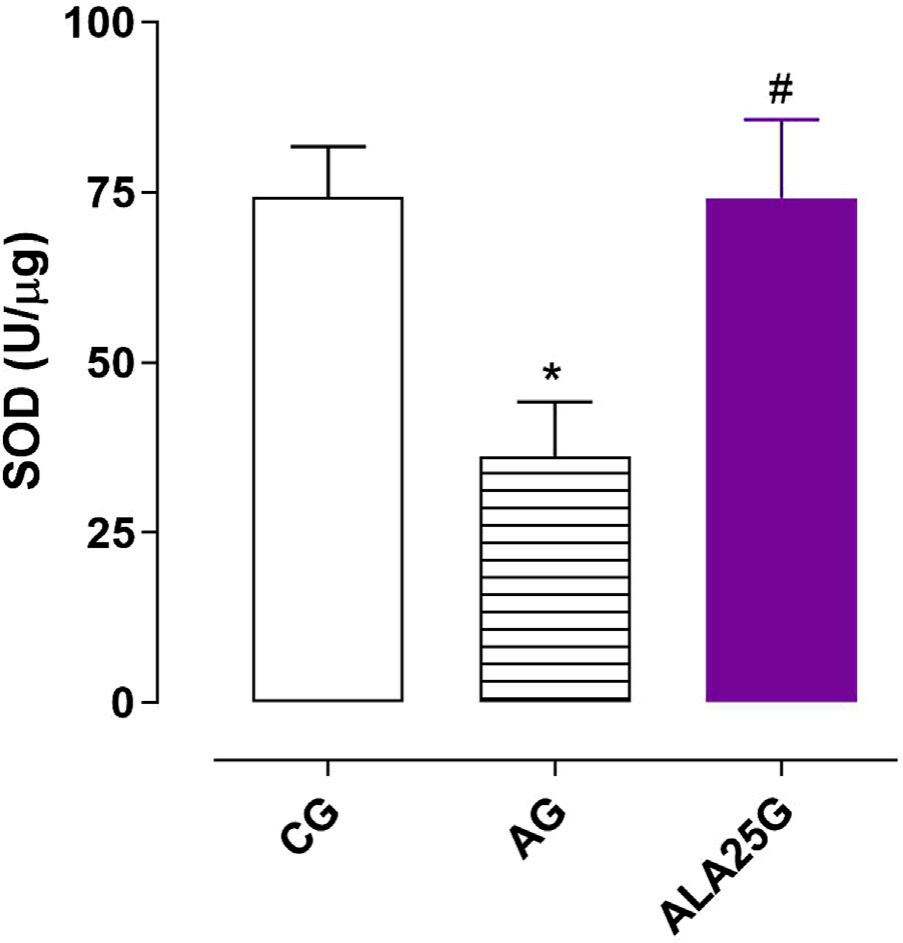
SOD activity (U/μg) in the lung homogenate. Symbols and vertical bars represent the mean and S.E.M., respectively. ANOVA one-way followed by Tukey’s *post hoc* test (n = 5). ^*^ *p* < 0.05 (CG vs. AG and ALA25G); ^#^ *p* < 0.05 (AG vs. ALA25G).

SOD activity was increased in the AG (36.2 ± 8.0 U/μg) compared to the CG (74.4 ± 7.3 U/μg). This increase was prevented when the animals were treated with lauric acid at a dose of 25 mg/kg (74.1 ± 11.6 U/μg) (Figure 10).

## 4 Discussion and conclusions

The present study demonstrated that lauric acid (LA) exerts its preventive effect on tracheal hyperresponsiveness by negatively modulating the cyclooxygenase and nitric oxide pathways, in addition to reducing oxidative stress imbalance, in an ovalbumin-induced allergic asthma model in Wistar rats.

Previous studies (Figueiredo et al., 2025) have shown the preventive effect of LA at doses of 25 mg/kg, 50 mg/kg, and 100 mg/kg on carbachol (CCh)-induced tracheal hyperresponsiveness and the reduction of aminophylline-induced relaxation, with the 25 mg/kg dose being the lowest effective dose in promoting these effects. Additionally, LA at 25 mg/kg was the only dose that prevented minute volume alterations observed in the AG. Thus, further investigation was carried out to elucidate the mechanism of action of LA at a dose of 25 mg/kg in the airway changes promoted by the OVA-induced allergic asthma model.

In asthma, an inflammatory infiltrate is observed in the peribronchovascular region, associated with increased mucus production, due to goblet cell metaplasia (Ma et al., 2021), which characterizes tissue remodeling in this region. Therefore, the preventive effect of LA on the anatomopathological findings of asthmatic rats was initially investigated. Tissue remodeling process in this asthma model was confirmed through anatomopathological analysis of the lung parenchyma, where a pronounced inflammatory infiltrate was observed in histological sections stained with HE (Figure 1). These alterations were prevented by treatment with different doses of LA and dexamethasone. Although it did not completely abolish inflammation, the reduction observed in ALA25G was sufficient to decrease tracheal hyperresponsiveness, according to previous results (Figueiredo et al., 2025).

Given that in previous *in silico* studies (Figueiredo et al., 2025), LA exhibited good binding affinity to various proteins related to airway contractility and remodeling, including COX-2, it was decided to investigate the involvement of this pathway in LA’s preventive mechanism of action.

COX metabolites have diverse effects on the lungs and are known to modify airway tone as well as inflammatory responses. For prostanoid generation, both COX-1, which is constitutively expressed by most cells, and COX-2, an inducible isoform upregulated by inflammatory mediators such as lipopolysaccharide, IL-1β, IL-6, or tumor necrosis factor-α (TNF-α), convert AA into prostaglandin endoperoxides (PGs), such as prostaglandin G_2_ (PGG_2_) (Ramsay et al., 2003; Ricciotti and Fitzgerald, 2011). Subsequently, an endoperoxidase reaction reduces PGG_2_ to PGH_2_, a highly unstable cyclic endoperoxide that is rapidly converted into bioactive prostanoids by specific synthases, including PGD_2_, PGE_2_, PGF_2α_, PGI_2_, and TxA_2_ (Zaslona and Peters-Golden, 2015).

Among the contractile prostaglandins of airway smooth muscle, PGD_2_ and PGF_2α_ stand out. Studies have already demonstrated an increase in prostanoids in the bronchoalveolar lavage fluid (BALF) of individuals with allergic asthma compared to healthy individuals, showing 12- and 22-fold increases in PGD_2_ and PGF_2α_ levels, respectively (Liu et al., 1990).

Thus, given that COXs are key enzymes involved in the release of mediators that either exacerbate or alleviate airway hyperresponsiveness in asthma, the participation of COX products was evaluated to determine whether these metabolites influence tracheal contractile responsiveness. For this purpose, indomethacin, a non-selective COX inhibitor (Hua et al., 1996), was used, and no change in contractile efficacy or potency in the presence of this inhibitor in the CG was observed (Figure 2; Table 1).

In the asthmatic animals, there was a reduction in contractile efficacy in the presence of indomethacin (approximately 82% reduction compared to the absence), without changes in potency, indicating that in this asthma induction model, inhibition of this enzyme resulted in reduced production of contractile prostanoids, such as PGD_2_ and PGF_2α_ (Figure 2; Table 1). Conversely, Vasconcelos et al. (2020) demonstrated that in the presence of indomethacin, the trachea of guinea pigs with OVA-induced allergic inflammation exhibited increased contractile efficacy. It can be inferred that methodological differences, such as the animal model, duration of ovalbumin exposure, and other factors, may account for these discrepancies. However, similar to the findings of the present study, Ferreira et al. (2025) observed a reduction in tracheal contractile efficacy, also without change in potency, in the asthmatic group of an OVA-induced asthma model in Wistar rats.

In the ALA25G, there was a reduction in tracheal contractile efficacy in the presence of indomethacin (close to 43% reduction compared to the absence), suggesting that LA may negatively modulate the cyclooxygenase pathway, further decreasing the production of contractile mediators (Figure 2; Table 1). These results align with findings from studies on supplementation with virgin coconut oil (VCO) in guinea pigs with pulmonary inflammation, where, in the presence of indomethacin, tracheal contractile efficacy and potency in response to CCh were not altered. This suggests that COX blockade by VCO would still allow sufficient production of relaxing prostanoids to reduce CCh-induced contractility (Vasconcelos et al., 2020), indicating that LA, the major component of VCO, may be responsible for this effect. A study by Henry et al. (2002) demonstrated that LA has inhibitory effects on both COX-1 and COX-2.

Another important enzyme in arachidonic acid metabolism is 5-lipoxygenase (5-LOX), which leads to the production of leukotrienes (Samuelsson et al., 1987). These are divided into two classes: LTB_4_ and cysteinyl leukotrienes (CysLTs) (Luginina et al., 2023). LTB_4_ has pro-inflammatory activity, triggering chemotaxis and subsequently activating the inflammatory response (Saeki and Yokomizo, 2017; Yokomizo and Shimizu, 2023).

LTC_4_, LTD_4_, and LTE_4_ constitute the CysLTs, which play a key role in the pathogenesis of asthma by inducing bronchoconstriction (O'Hickey et al., 1991; Lee et al., 2024), promoting tissue remodeling (Henderson Jr et al., 2006; Mehrotra and Henderson Jr, 2009), and increasing inflammation through the recruitment of eosinophils, mast cells, T lymphocytes, monocytes, and basophils, in addition to stimulating the production of Th2 cytokines (Kim et al., 2006; Montuschi, 2010). Studies report that CysLT and LTB_4_ levels in bodily fluids (sputum, BAL, serum, and urine) of asthmatic patients are significantly higher than those in healthy individuals and increase with asthma severity (Kazani et al., 2013; Uchida et al., 2019).

Thus, the present study evaluated the effect of the 5-LOX pathway on the mechanisms underlying the changes caused by asthma and LA. For this purpose, zileuton, a 5-LOX inhibitor (Malo et al., 1994), was used as a pharmacological tool. It was observed that in the CG, the presence of this inhibitor did not alter contractile efficacy or potency. Conversely, in the AG, there was a reduction in contractile efficacy (almost 60% reduction, compared to the absence) but not in potency in the presence of the inhibitor, indicating that the increased tracheal hyperresponsiveness in AG may be due to the high production of CysLTs (Figure 3; Table 2). These findings are consistent with previous literature demonstrating the role of 5-LOX inhibition in reducing airway reactivity (Malo et al., 1994; Irvin et al., 1997).

On the other hand, LA does not negatively modulate the 5-LOX pathway, as no differences in contractile reactivity to CCh were observed in the ALA25G in the presence of zileuton. These results are in line with *in silico* studies by Figueiredo et al. (2025), in which LA, despite presenting a negative binding energy with 5-LOX, was not found to be more favorable than zileuton.

Another important airway mediator is nitric oxide (NO^·^), which is produced by different isoforms of nitric oxide synthase (NOS), including nNOS, eNOS, and iNOS. This liposoluble gas activates soluble guanylyl cyclase (sGC), which cleaves and cyclizes GTP into cyclic guanosine monophosphate (cGMP). cGMP then activates cGMP-dependent kinase (PKG), leading to smooth muscle relaxation (Zhao et al., 2015).

There is an increased expression of iNOS in the airways of asthmatic individuals, particularly in epithelial and inflammatory cells, including macrophages, neutrophils, and eosinophils. This increased and/or expression of iNOS activity occurs due to induction by inflammatory cytokines, and this isoform is responsible for producing large amounts of NO^·^ (Yan et al., 1995; Ricciardolo et al., 2004). High NO^·^ levels have been associated with airway hyperresponsiveness due to the formation of the free radical peroxynitrite (ONOO^−^) (Sadeghi-Hashjin et al., 1998).

Thus, it was decided to investigate whether the tracheal hyperresponsiveness to CCh in asthmatic rats was associated with the NO^·^ pathway and whether lauric acid exerts its preventive effect by modulating this pathway. To this end, L-NAME, a NOS inhibitor (Sousa et al., 2010), was used, and it was observed that the contractile response to CCh in the CG was not altered in the presence of this inhibitor. Because NO^·^ has a bronchodilator effect, it would be expected that inhibiting its synthesis with L-NAME would increase CCh-induced contraction. However, this effect was not observed in the present study for the CG, suggesting that in non-asthmatic animals, NO^·^ does not exert significant tonic control over airway contractility (Figure 4; Table 3).

In the asthmatic group (AG), a reduction in contractile efficacy to CCh was observed in the presence of L-NAME (38% reduction, compared to the absence) (Figure 4; Table 3), indicating that in this asthma induction model, there is an exacerbated formation of NO^·^, which consequently reacts with the superoxide anion ^·^O_2_^−^ to form ONOO^−^, responsible for the hyperresponsiveness of asthmatic airways (Sadeghi-Hashjin et al., 1998).

Different results were observed in the trachea of guinea pigs with chronic pulmonary inflammation in the presence of L-NAME, where an increase in both contractile efficacy and potency to CCh was noted. This suggests that in this airway inflammation model, L-NAME exhibits a different activity on NOS isoforms, presumably blocking eNOS but not iNOS (Vasconcelos et al., 2020).

In the ALA25G, the contractile response to CCh in the presence of L-NAME was also reduced (40%, compared to the absence) (Figure 4; Table 3), suggesting a negative modulation of NOS by LA. These data align with the findings from *in silico* analyses of LA, where this fatty acid showed better binding affinity to both eNOS and iNOS isoforms than to their respective inhibitors (Figueiredo et al., 2025). In addition, LA has been shown to reduce iNOS activity in the lungs of type II diabetic Wistar rats (Augustine et al., 2022).

Asthma is characterized by an oxidative stress imbalance, caused by an overload of oxidant species and a reduction in antioxidant defenses (Sahiner et al., 2018). Among the reactive oxygen species (ROS), free radicals with unpaired electrons, such as the superoxide anion (^·^O_2_^−^), hydroxyl radical (HO^·^), hydroperoxyl radical (HO_2_^·^), and peroxyl radical (RO_2_^·^), as well as non-radical oxygen derivatives like hydrogen peroxide (H_2_O_2_), hypochlorous acid (HClO), and ozone (O_3_), can be mentioned. Among reactive nitrogen species (RNS), nitric oxide (NO^·^) and nitrogen dioxide (NO_2_^·^) are notable, as well as non-radical species such as nitrite (NO_2_^−^) and peroxynitrite (ONOO^−^) (Sies et al., 2017).

An important role is attributed to NADPH oxidase, which is responsible for the formation of ^·^O_2_^−^ through electron transfer from NADPH. This compound can be spontaneously or enzymatically dismutated to H_2_O_2_ (Taylor and Hubert, 2021). Studies report that these ROS induce contraction of the guinea pig trachea both directly (Rhoden and Barnes, 1989) and by influencing airway reactivity to contractile agonists, such as acetylcholine and methacholine (Kudo et al., 1996; Nishikawa et al., 1996; Henricks and Nijkamp, 2001), and on electrical field stimulation-induced contractile responses of isolated rat intrapulmonary bronchi (Szarek and Schmidt, 1990).

Cells have antioxidant enzymatic systems to regulate homeostasis in the formation of reactive species in the airways, such as the superoxide dismutase (SOD) complex, which converts ^·^O_2_^−^ into H_2_O_2_; catalase, which converts H_2_O_2_ into water and O_2_; and glutathione peroxidase (GPx) and peroxiredoxin, which inactivate H_2_O_2_ and other hydroperoxides (Pietarinen-Runtti et al., 2000; Wang et al., 2023). Additionally, there are non-enzymatic antioxidant systems, including reduced glutathione (GSH), vitamins (C and E), and minerals (selenium and zinc) (Sies et al., 2022).

Initially, the participation of the superoxide anion produced by NADPH oxidase in CCh-mediated contraction in the rat trachea was evaluated. For this, apocynin, a blocker of this enzymatic complex (Shabir et al., 2014), was used as a pharmacological tool. It was observed that there was no difference in contractile efficacy or potency in the CG in the presence of this inhibitor (Figure 4; Table 4). Conversely, because the inhibition of NADPH oxidase generates a lower amount of ^·^O_2_^−^, a reduction in contractile efficacy was observed in the AG in the presence of apocynin (nearly 67%, compared to the absence), indicating that in this asthma model, ^·^O_2_^−^ production appears to contribute to oxidative stress and increased airway hyperresponsiveness. A similar result was observed by Ferreira et al. (2025) in an ovalbumin-induced asthma model in Wistar rats.

In the ALA25G group, a reduction in contractile efficacy in the presence of apocynin, compared to its absence (27% reduction), was observed, without changes in potency (Figure 5; Table 4). This suggests a negative modulatory effect on NADPH oxidase or the production of reactive species, indicating that LA may improve the oxidative stress imbalance. These results align with previous findings, as it was suggested that LA may reduce the formation of peroxynitrite (ONOO^−^) by negatively modulating iNOS. Complementarily, this reduction in ONOO^−^ production could also occur through a decrease in the production of ^·^O_2_^−^. Another study demonstrated the reduction of NADPH oxidase-derived ^·^O_2_^−^ production by LA in the heart and kidneys of spontaneously hypertensive rats (Alves et al., 2017).

Similar results were observed in the presence of tempol, a SOD mimetic (De Boer et al., 2001). No difference in contractile efficacy or potency was observed in the CG in the presence of this inhibitor (Figure 6; Table 5). However, the reduction in contractility observed in the presence of tempol in the AG (44% reduction, compared to the absence) may be due to the conversion of ^·^O_2_^−^ into H_2_O_2_ stimulated by this mimetic, thereby reducing the amount of ^·^O_2_^−^ and its availability for ONOO^−^ formation, which is produced through its reaction with NO^·^, ultimately decreasing tracheal hyperresponsiveness.

In the ALA25G, contractile efficacy or potency was not altered in the presence of tempol (Figure 6; Table 5). Nevertheless, based on the previously presented data, it is suggested that LA negatively modulates ^·^O_2_^−^ formation, leading to insufficient substrate available for conversion into H_2_O_2_, which justifies the absence of changes in tracheal contractility in the presence of tempol. LA may stimulate antioxidant defenses to neutralize these ROS.

Increased ROS production can trigger chain reactions of lipid peroxidation, leading to the formation of unstable lipid radicals (Cordiano et al., 2023; Wang et al., 2023). Malondialdehyde (MDA) is the primary and most widely studied product of lipid peroxidation, commonly used as a measure of oxidative stress (Ayala et al., 2014; Barrera et al., 2018). MDA can covalently interact and cause damage to membrane proteins, nucleic acids, or adjacent polyunsaturated fatty acids (Marnett, 1999; Ayala et al., 2014).

Thus, oxidative stress balance and antioxidant defenses in the pulmonary homogenate of rats were evaluated to support the functional data observed. Initially, lipid peroxidation levels were quantified by measuring MDA production, and an increase in these levels was observed in the AG compared to the CG (Figure 7). These data align with other studies on OVA-induced asthma models in mice, which also reported an increase in MDA levels in the AG (Nesi et al., 2017; Bao et al., 2018; Hanna et al., 2019; Jasemi et al., 2022).

Treatment with lauric acid at a dose of 25 mg/kg prevented the increase in lipid peroxidation observed in the AG, supporting the data indicating that LA mitigates the oxidative stress imbalance (Figure 7). Other studies have already reported the reduction of MDA levels promoted by LA in the rat liver with non-alcoholic fatty liver disease (Sedik et al., 2024), with ethanol-induced hepatotoxicity (Namachivayam and Gopalakrishnan, 2023), and in the serum, testes, and epididymis of diabetic rats (Anuar et al., 2023).

Another marker of oxidative stress is nitrite (NO_2_^−^), a product of NO^·^ metabolism in the presence of molecular oxygen, which is widely reported in high concentrations in the exhaled air of asthmatic individuals (Hunt et al., 1995; Formanek et al., 2002; Ueno et al., 2008; Rihák et al., 2010). NO_2_^−^ is a substrate for the enzymes myeloperoxidase and eosinophil peroxidase, resulting in the formation of the nitrogen dioxide radical (NO_2_^·^) (Ghosh and Erzurum, 2012). NO_2_^·^, along with ONOO^−^, is responsible for the nitration of tyrosine residues in proteins and the formation of 3-nitrotyrosine, which is extensively found in the lungs of asthmatic individuals (Duguet et al., 2001; Dweik et al., 2001; Andreadis et al., 2003; Zuo et al., 2014).

An increase in nitrite levels was observed in the pulmonary homogenate of asthmatic animals compared to the CG, which was prevented in the ALA25G (Figure 8). These data are consistent with those previously obtained, suggesting that by reducing the exacerbated production of NO^·^ through the inhibition of iNOS, LA also decreases the metabolism of NO^·^ to nitrite.

The levels of GSH, a non-enzymatic antioxidant, and the activity of SOD were quantified in the analysis of the effect of alterations and LA on antioxidant systems. GSH reduces organic hydroperoxides, protecting against lipid peroxidation. It is oxidized through a reaction involving glutathione peroxidase (GPx), forming glutathione disulfide (GSSG) (Barnabas et al., 2023).

Studies report reduced levels of GSH and the GSH/GSSG ratio in individuals with allergic asthma (Ercan et al., 2006; Sackesen et al., 2008; Deveci et al., 2004) and show that the use of GSH precursors contributes to the reduction of inflammation and hyperresponsiveness in an ovalbumin-induced allergic asthma model in mice (Koike et al., 2007). These data are consistent with the findings of the present study, as a reduction in GSH levels was observed in the AG, compared to the CG (Figure 9). This reduction was not prevented in ALA25G, suggesting that LA exerts its effect in reducing oxidative stress imbalance through mechanisms that do not involve an increase in this antioxidant. Preventive effect of LA in reducing GSH levels has also been reported in the rat liver with ethanol-induced hepatotoxicity (Namachivayam and Gopalakrishnan, 2023).

*In vitro* studies have shown that reactive oxygen and nitrogen species lead to oxidative and nitrative modification of tyrosine and inactivation of superoxide dismutases (SOD) (MacMillan-Crow and Thompson, 1999; Alvarez et al., 2004). Therefore, the reduction in SOD activity may be associated with inflammation and airway hyperresponsiveness in asthmatic individuals (Sugiura and Ichinose, 2008).

Similar to what was observed for GSH, SOD activity was also reduced in the AG compared to the CG (Figure 10), supporting the findings in tracheal reactivity in the presence of tempol, a SOD mimetic, where a reduction in contractile efficacy was observed. This suggests that tempol is likely compensating for SOD activity, given that this enzyme’s function is impaired in this group.

The reduction in SOD activity in the AG was prevented when animals were treated with lauric acid at a dose of 25 mg/kg (Figure 10), suggesting that AL may exert an antioxidant effect by positively modulating this enzyme’s activity. These findings align with previous observations, where contractile reactivity to CCh was not altered in the ALA25G in the presence of tempol compared to its absence (Figure 6; Table 5). This indicates that, as AL decreases ^·^O_2_^−^ production, the lower availability of this ROS allows it to be metabolized by SOD, whose activity reduction was prevented by LA, thereby preventing tempol from exerting its action. The preventive effect of LA in reducing SOD activity has also been reported in the rat liver with ethanol-induced hepatotoxicity and an ischemic brain (Namachivayam and Gopalakrishnan, 2023; Shaheryar et al., 2023).

Based on the results obtained, it can be concluded that lauric acid reduces tracheal hyperresponsiveness in Wistar rats with allergic asthma by negatively modulating both the COX and NO pathways, as well as the imbalance of oxidative stress. Thus, it is suggested that the pharmacological effects of coconut oil in preventing asthma-associated alterations in murine models may be, at least in part, attributed to the action of lauric acid. Further studies may be carried out to complement the mechanism of action of lauric acid.

## Data Availability

The raw data supporting the conclusions of this article will be made available by the authors, without undue reservation.
